# GABAergic Neurons as Putative Neurochemical Substrate Mediating Aversive Effects of Nicotine

**DOI:** 10.4172/2329-6488.1000312

**Published:** 2018-04-30

**Authors:** Ozra Dehkordi, Jed E. Rose, Richard M. Millis, Maryam Mehdipour Dalivand, Shereé M. Johnson

**Affiliations:** 1 Department of Neurology, Howard University Hospital Washington D.C. 20060, United States; 2 Department of Physiology & Biophysics, Howard University College of Medicine Washington, D.C. 20059, United States; 3 Department of Psychiatry, Duke University Medical Center, Durham, NC 27705, United States; 4 Department of Medical Physiology, American University of Antigua College of Medicine, Antigua & Barbuda, West Indies

**Keywords:** Addiction, GAD67-GFP, GABAergic, Ventral Tegmental Area (VTA)

## Abstract

Nicotine, the main addictive component of tobacco smoke, has both rewarding and aversive properties. Recent studies have suggested that GABAergic neurons, one of the main neurochemical components of the reward-addiction circuitry, may also play a role in the aversive responses to nicotine. In the present study of transgenic mice expressing Green Fluorescent Protein (GFP) in Glutamate Decarboxylase 67 (GAD67) neurons, we hypothesized that a subpopulation of GABAergic neurons in the Ventral Tegmental Area (VTA) are the targets of aversive doses of nicotine in the CNS. We tested this hypothesis using c-Fos immunohistochemical techniques to identify GAD67-GFP positive cells within the VTA, that are activated by a single intraperitoneal (i.p.) injection of a low (40 ug/kg) or a high (2 mg/kg) dose of nicotine. We also assessed the anatomical location of GAD67-GFP positive cells with respect to tyrosine hydroxylase (TH) Immunoreactive (IR) dopaminergic cells in VTA. Consistent with our previous studies low- and high-dose nicotine both induced c-Fos activation of various intensities at multiple sites in VTA. Double labeling of c-Fos activated cells with GAD67-GFP positive cells identified a subpopulation of GABAergic neurons in Substantia Nigra Compact part Medial tier (SNCM) that were activated by high- but not by low-dose nicotine. Of 217 GABAergic cells counted at this site, 48.9% exhibited nicotine induced c-fos immunoreactivity. GAD67-GFP positive cells in other regions of VTA were not activated by the nicotine doses tested. Double labeling of GAD67-GFP positive cells with TH IR cells showed that the GABAergic neurons that were activated by high-dose nicotine were located in close proximity to the dopaminergic neurons of substantia nigra compact part and VTA. Dose-dependent activation of GAD67-GFP positive neurons in SNCM, by a nicotine dose known to produce aversive responses, implies that GABAergic neurons at these sites may be an important component of the nicotine aversive circuitry.

## Introduction

Nicotine, the main addictive component of cigarette smoke, acts on various nicotinic acetylcholine receptors (nAChRs) of the mesolimbic reward pathways to facilitate dopamine (DA) release in nucleus accumbens [1–5]. The precise mechanism by which nicotine acts to regulate the activity of DA signaling is not clear. Multiple biochemical studies have demonstrated that the activity of dopaminergic neurons in ventral tegmental area (VTA) is regulated by glutamatergic and GABAergic interneurons, as well as by projections from other brain regions [6–8]. GABAergic and glutamatergic synaptic inputs to the DA neurons of VTA are modulated by various nAChRs, which possess distinct desensitization properties [8]. Electrophysiological studies have shown that a single exposure to nicotine causes a transient increase in GABAergic transmission [8]. This response is then followed by a persistent depression of the inhibitory GABAergic signal, caused by desensitization of the nAChRs [8–10]. Concurrently, nicotine enhances glutamatergic transmission, via nAChRs which desensitize less than the nAChRs on GABAergic neurons, thereby, shifting the balance toward excitation [8,11–13].

We previously demonstrated that a single intraperitoneal (i.p.) injection of nicotine activates multiple cells in VTA, as well as other structures of the reward-addiction circuitry [14–16]. The neurochemical profile of neurons that are the initial target of acute nicotine is not known. Dopaminergic neurons that are activated by chronic nicotine do not appear to be the initial target of a single injection of nicotine in the VTA [15]. In the present study, we determined whether GABAergic neurons, which are implicated in both reward and aversive responses to nicotine, are potential targets of an acute single injection of nicotine in the VTA [17–20]. We evaluated the effects of two different doses of nicotine, one that is non-aversive [21,22] and the other within the range that produces aversive responses in mice [21,22]. We also assessed the anatomical location of GABAergic neurons with respect to the dopaminergic cells of VTA.

## Materials and Methods

### Subjects

Adult (2–3 month-old) CD-1 GAD67-GFP transgenic mice weighing 20–25 g were used. In the GAD67-GFP mice, GABAergic neurons co-express Green Fluorescent Protein (GFP) with the native protein GAD67 allowing these neurons to be easily identified with the use of fluorescent microscopy [23]. All procedures including the anaesthesia and surgery were approved by the Institutional Animal Care and Use Committee (IACUC) of Howard University. All efforts were made to minimize the number of animals used and their suffering.

### Experimental protocol

Animals (N=15) were housed at a room temperature of 22–24°C with water and food freely available. To reduce the nonspecific effects of handling and experimental environment, animals were handled daily and exposed to the same conditions as during the actual experiments. Following an adaptation period of 3–4 d, the mice were treated by i.p. injection of saline (control), and/or nicotine hydrogen tartrate salt (Sigma-Aldrich, Saint Louis, MO). The initial nicotine dose (40 μg/kg) used in the present study is non-aversive and comparable to the dose delivered during the smoking of one or two cigarettes in humans [21,22,24]. This dose is also within the range reported to induce c-Fos activations at multiple brain regions in mice [14]. The high dose of nicotine (2 mg/kg) used in the present study is within the range that has been shown to produce aversive responses in mice [21,22]. Both doses of nicotine were dissolved in saline and injected i.p. in volumes of 0.2 ml/injection. Two h after i.p. injection of the saline (control) and/or the nicotine, the mice were anesthetized with 5% isoflurane and were perfused transcardially with saline, followed by 4% paraformaldehyde in 0.1 M Phosphate Buffer (PB) at pH 7.4. After perfusion, the brains were postfixed in 4% paraformaldehyde for one h and then cryoprotected in a 30% sucrose solution for a minimum of 2 d. Transverse sections of the brain were cut at 40 μm using a Bright OTF Cryostat (Hacker Instruments and Industries) and were stored in 0.5% sodium azide in 0.1 M PB (pH 7.4).

### Immunohistochemistry

Immunohistochemical procedures were performed using free floating sections as follows: Briefly, 1-in-5 series of brain sections extending from bregma-3.87 mm to bregma-2.79 mm [25] were rinsed three times in 0.1 M Phosphate Buffered Saline (PBS) at pH 7.4. Nonspecific binding was blocked by incubating the tissues overnight in loading buffer containing 2% Normal Donkey Serum (NDS, Santa Cruz Biotechnology, Inc., Santa Cruz, CA) and 0.3% Triton X-100. Tissues were then washed and processed for sequential double labeling of GAD67-GFP positive cells with nicotine-induced c-Fos, physiological saline-induced c-Fos, or Tyrosine Hydroxylase (TH) Immunoreactive (IR) cells according to the following protocols.

### Double labeling of GAD67-GFP positive cells and TH-IR dopaminergic cells

For double labeling of TH with GAD67-GFP positive GABAergic neurons, tissues were washed and incubated with a PBS cocktail consisting of 0.3% Triton X-100 and mouse anti-TH (1:1000; Cat #T1299, Sigma-Aldrich) antibodies at 4°C for 48 h. After washing in PBS, sections were incubated with Alexa Fluor 594 donkey anti-mouse secondary antibody (1:100, Jackson Immuno-Research Laboratories, Inc.) for 2½ h. Finally, the sections were rinsed in PBS and cover-slipped using Vecta Shield (Vector Laboratories) anti-fade mounting media.

### Double labeling of GAD67-GFP positive cells and nicotine induced c-Fos IR cells

For double labeling of nicotine-induced c-Fos and GAD67-GFP containing GABAergic neurons, tissues were washed and incubated with a PBS cocktail consisting of 0.3% Triton X-100 and rabbit anti-c-Fos (1:1000, Millipore Corporation, Temecula, CA) at 4°C for 48–72 h. The sections were then incubated in Alexa Fluor 594 donkey anti-rabbit secondary antibody (1:100; Jackson ImmunoResearch Laboratories, Inc.) in 0.1 M PBS for 2½ h. After washing in PBS, sections were rinsed in PBS, mounted and cover-slipped using Vecta Shield (Vector Laboratories Inc., CA) anti-fade mounting media.

Controls for each experiment were performed to determine whether the primary or the secondary antibodies produced false-positive results. The controls involved omission of the primary and/or secondary antisera to eliminate the corresponding specific labeling. Nonspecific activation of c-Fos was assessed by evaluating the CNS expression of c-Fos in animals receiving i.p. injection of normal Physiological Saline (PS).

### Data analysis

High-resolution fluorescent images were acquired using Nikon (Nikon Instruments, Melville, NY) and Olympus (Olympus AX70, Olympus America) microscopes equipped with the adequate filter systems to observe the red and green fluorescence. Co-localization of GAD67 GFP-containing cells with c-Fos and/or TH-IR dopaminergic cells was detected by sequential capturing of the images, alternating between filters appropriate for each labeling and by analyzing the merged images of the exact same sites. Images from all the brain regions of interest were captured at 4×, 10× and 20× magnification and minor adjustments of brightness and contrast were made using Adobe Photoshop CS3.

A semi-quantitative estimate of the total number of GAD67-GFP positive cells in VTA that were activated by nicotine induction of c-Fos was performed as follows: Representative 40 μm sections from regions of interest were selected for each animal in the group (N=4). The total numbers of cells that exhibited GAD67-GFP and nicotine-induced c-Fos were counted, and the percentage of GAD67-GFP positive cells that expressed c-Fos immunoreactivity was computed.

## Results

### Localization of GAD67-GFP positive cells with respect to the TH positive dopaminergic neurons of VTA

**Figure 1:**
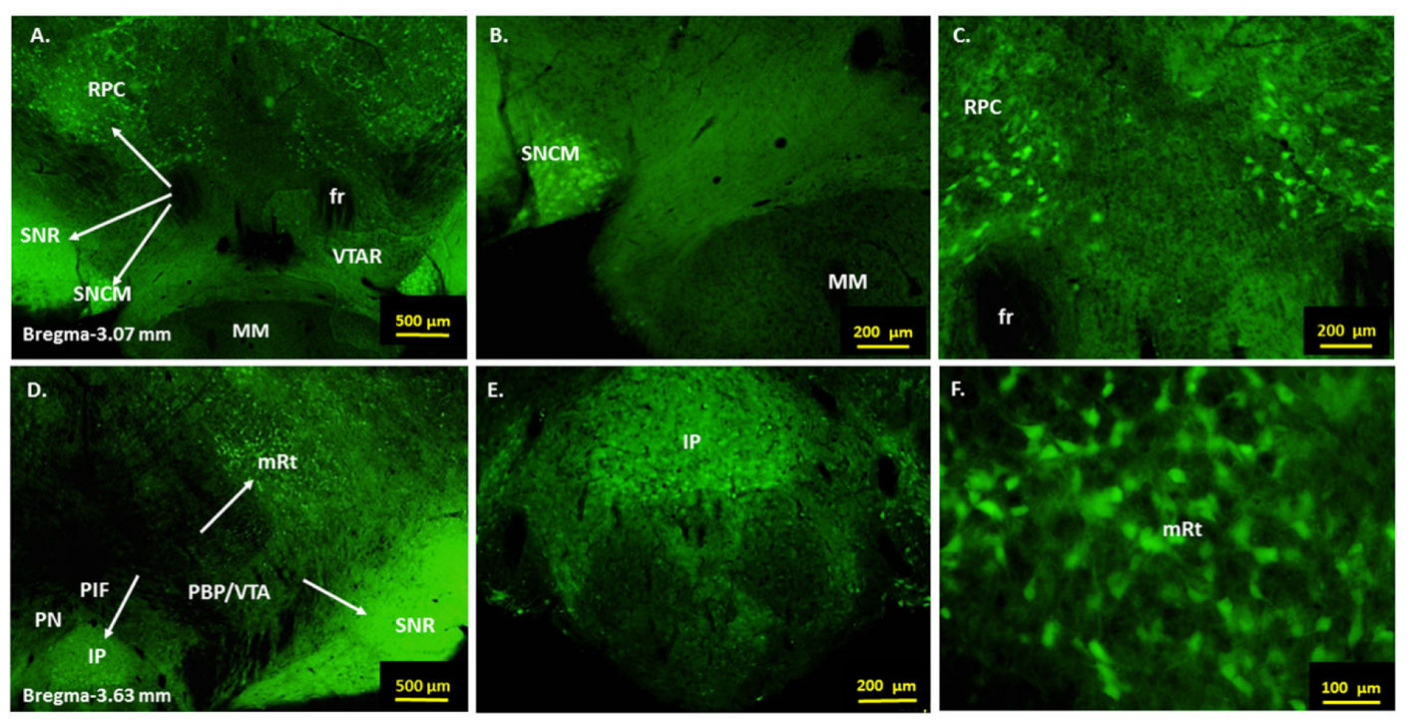
Anatomical location of GAD67-GFP positive GABAergic neurons in rostral and caudal ventral tegmental area (VTA). Panels A–C: Low and high power fluorescent images showing GAD67-GFP positive cells in the rostral regions of VTA. Panels D–F: Low and high power fluorescent images showing GAD67-GFP positive cells in the caudal regions of VTA. Arrows indicate locations of GAD67-GFP positive cells. GABAergic neurons are seen at multiple sites of VTA. Abbreviations: VTAR= ventral tegmental area rostral, SNR= substantia nigra reticular part, SNCM= substantia nigra compact part, medial tier, RPC= red nucleus parvocellular part, PN= paranigral nucleus, PBP= parabrachial pigmented nucleus, PIF= parainterfascicular nucleus, IP= interpeduncular nucleus, mRt= mesencephalic reticular formation, MM= medial mammillary nucleus, fr= fasciculus retroflexus.

GAD67-GFP positive GABAergic neurons were observed at multiple sites of the mesolimbic reward-addiction pathways. In rostral VTA, corresponding to bregma −3.15 to −3.07 mm, GAD67-GFP positive cells were seen in areas overlapping Substantia Nigra Reticular part (SNR), Substantia Nigra Compact part, Medial tier (SNCM) and Red nucleus Parvocellular part (RPC) (Figure 1: Panels A–C). In caudal VTA, corresponding to bregma −3.87 to −3.63 mm, GAD67-GFP positive cells were seen in areas that were ventral, lateral and rostral to Paranigral Nucleus (PN), Parabrachial pigmented nucleus (PBP) and to Parainterfascicular Nucleus (PIF) of VTA and at sites that correspond to Interpeduncular Nucleus (IP), SNR and Mesencephalic reticular formation (mRt), respectively (Figure 1: Panels D–F).

**Figure 2:**
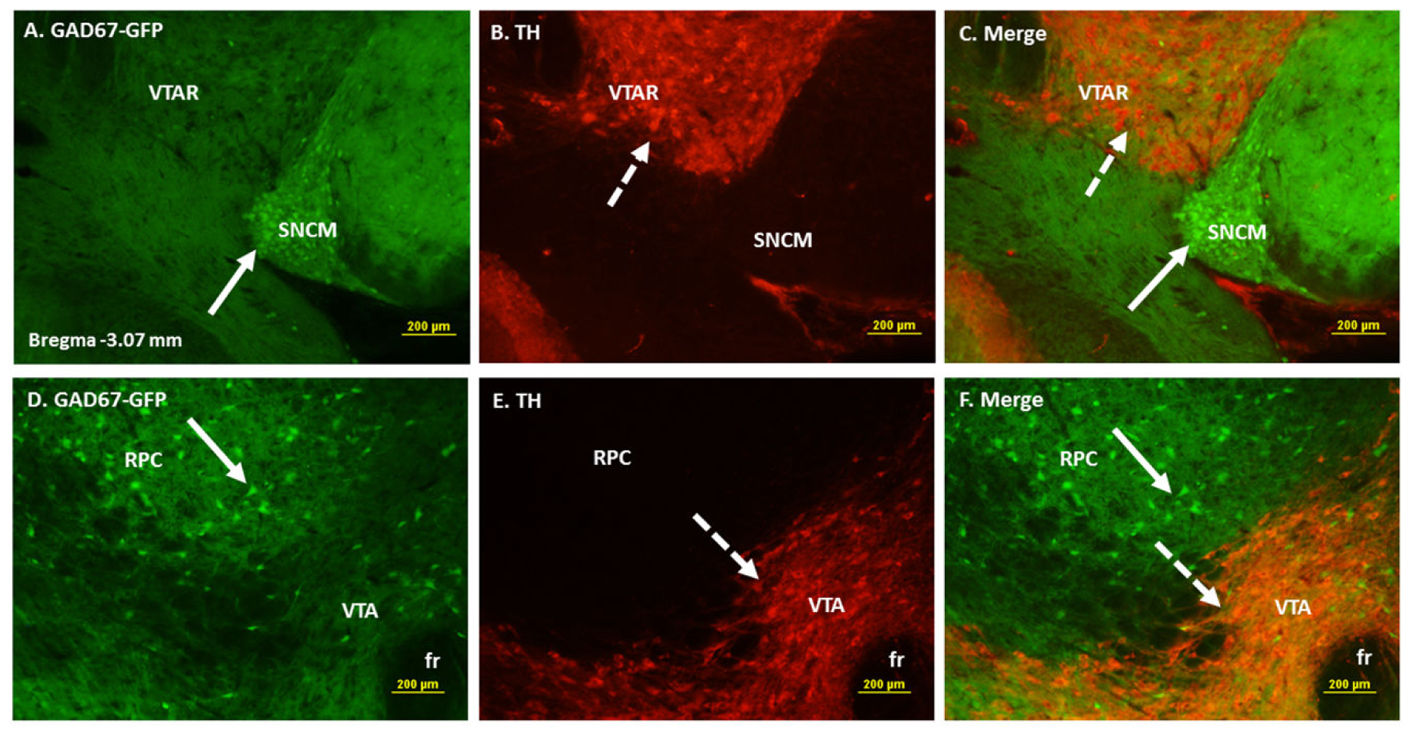
Double immunofluorescence labeling demonstrating the location of GAD67-GFP positive cells with respect to the tyrosine hydroxylase (TH) immunoreactive (IR) dopaminergic neurons of ventral tegmental area (VTA). Panels A–C: High power images showing GAD67-GFP positive cells (A), TH IR cells (B) and merged images of GAD67-GFP and TH in areas overlapping VTA, SNC and SNCM. Panels D–F: High power images showing GAD67-GFP positive cells (D), TH IR cells (E) and merged images of GAD67-GFP areas overlapping VTA and RPC. GABAergic neurons are seen in close proximity to the dopaminergic neurons of VTA. Solid arrows point to representative GAD67-GFP positive GABAergic cells and broken arrows to TH IR dopaminergic cells. Abbreviations: VTAR= ventral tegmental area rostral, SNCM= substantia nigra compact part, medial tier, fr= fasciculus retroflexus, RPC= red nucleus parvocellular part.

TH positive dopaminergic cells were identified at several sites in close proximity to the GAD67-GFP positive cells of VTA. In rostral VTA, dopaminergic neurons were found lateral to the GABAergic cells of SNR and SNCM (Figure 2: Panels A–C). GAD67-GFP positive cells were also observed dorsal to dopaminergic cells and in areas overlapping RPC (Figure 2: Panels D–F). In caudal VTA, TH positive dopaminergic neurons were found dorsal to the GAD67 positive GABAergic cells of IP and lateral to the GABAergic cells in SNR and mRt (data not shown).

### Co-expression of nicotine-activated cells and GAD67-GFP positive cells in VTA

**Figure 3:**
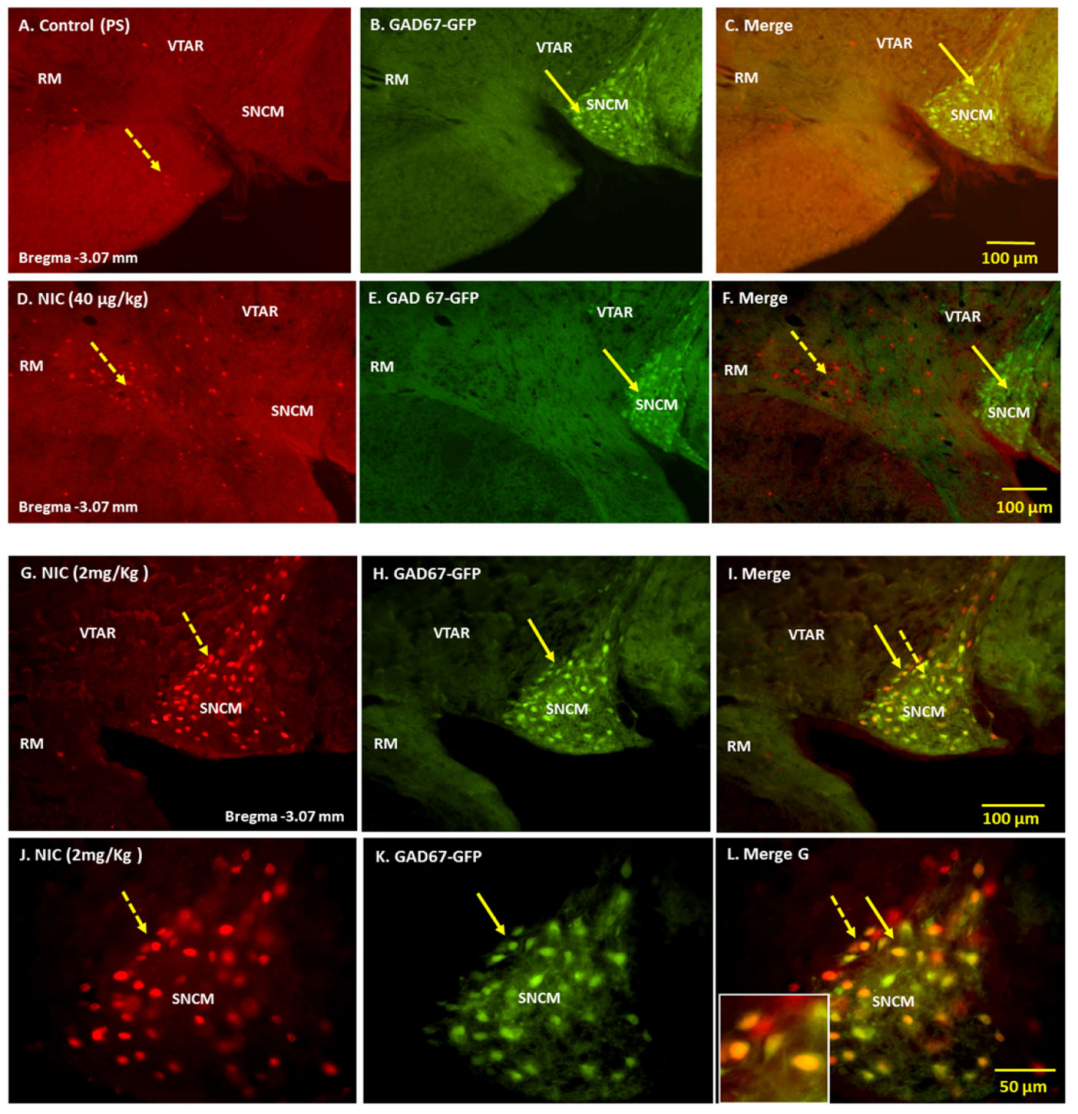
Double immunofluorescence labeling demonstrating the location of nicotine induced-c-Fos activated cells with respect to GAD67-GFP positive cells in ventral tegmental area (VTA). Panels A–C: Physiological saline (PS, control) induced c-Fos activated cells (A), GAD67-GFP positive cells (B) and merged images of PS and GAD67-GFP. Panels D–F: Cells activated by low-dose nicotine (D), GAD67-GFP positive cells (E), and merged images of nicotine-activated cells and GAD67-GFP positive cells. Panels G–I: Cells activated by high-dose nicotine (G), GAD67-GFP positive cells (H) and merged images of nicotine-activated cells and GAD67-GFP positive cells. Panels J–L: Higher magnification of panels G, H and I, respectively. High-dose nicotine activated a subpopulation of cells in SNCM. Solid arrows point to GAD67-GFP positive cells and broken arrows to nicotine-induced c-Fos-activated cells. The square in Panel L represents a magnified area showing expression of c-Fos in GAD67-GFP positive cells. Abbreviations: SNCM= substantia nigra compact part, medial tier, RM=retromamillary nucleus, VTAR= ventral tegmental area rostral.

Physiological saline (PS) induced c-Fos activation in a scarce number of cells in VTA. (Figure 3: Panels A–C). Consistent with our previous studies [14–15], acute single injection of low doses of nicotine (40 μg/kg) induced c-Fos expression in various structures of the mesocorticolimbic system. In the rostral regions of VTA, nicotine-activated cells were detected in areas ventral to the GAD67-GFP positive cells of RPC and lateral to the GABAergic cells of SNR and SNCM (Figure 3: Panels D–F). This dose of nicotine did not induce c-Fos activation in the GAD67-GFP positive cells of rostral VTA. In caudal VTA, the nicotine-activated cells, although scarce, were found dorsal to the GAD67-GFP positive cells of IP and medial to those of SNR. Although few c-Fos IR cells were detected in IP, these cells were distinct from the GABAergic neurons of IP and did not exhibit GAD67-GFP immunoreactivity (data not shown).

In contrast to low-dose nicotine (40 μg/kg), high-dose nicotine (2.0 mg/kg) selectively activated a subpopulation of GAD67-GFP positive cells in rostral VTA in areas overlapping SNCM (Figure 3: Panels G–L). Among 217 GAD67-GFP positive cells counted in representative midbrain sections overlapping SNCM, 47.9% exhibited immunoreactivity for c-Fos. The GABAergic neurons in other areas of VTA were not activated by either the low or the high dose of nicotine, under these experimental conditions.

## Discussion

The present study provides the first neuroanatomical data demonstrating that an acute single dose of nicotine activates a subpopulation of GABAergic cells in the vicinity of dopaminergic neurons of VTA and substantia nigra. The effect of nicotine on GABAergic cells at this site was dose-dependent and only seen following high-dose nicotine. These anatomical findings support previous electrophysiological data indicating that nicotine, acting through nAChRs, enhances GABAergic neurotransmission in multiple brain regions, including VTA [8,26].

In addition to the local GABAergic interneurons, VTA DA neurons receive GABAergic inputs from multiple other brain regions including projection fibers from the nucleus accumbens (Acb) and the ventral pallidum [6,7,27]. Although we were not able to detect nicotine activation of GABAergic cells in other brain regions, the possibility that higher doses of nicotine may recruit GABA-containing neurons in other regions of the nicotine reward-addiction and/or aversive circuitries cannot be ruled out.

Consistent with our previous studies, many of the areas activated by low-dose nicotine were also targets of high-dose nicotine [14–16]. Whether the same neurons are targets of both high- and low-dose nicotine at these sites is not clear. Our results demonstrating dose-specific responses of GABAergic neurons to nicotine in SNCM imply that different neurons and neurocircuitry may mediate the pharmacological effects of high versus low doses of nicotine in the CNS. Indeed, previous studies have suggested that different nAChR populations might mediate the pharmacological effects of higher versus lower doses of nicotine [28].

Nicotine is known to produce both rewarding and aversive responses [21,29,30]. The CD-1 mice used in the present study are known to exhibit both nicotine-conditioned place preference (CPP) and nicotine-conditioned taste aversion (CTA) in a dose-related manner [21]. How GAD67-GFP positive cells of SNCM, contribute to the abuse-related aspect of nicotine is not known. Behavioral studies have demonstrated that the nicotine dose, which we have shown to activate GABAergic cells of SNCM, is within the range that also produces CTA [21,22,31]. Additionally, studies have reported that the α4 subunit preferring antagonist, Dihydro-β-Erythroidine (DHβE) [32] attenuates nicotine-induced CTA, thereby suggesting that the aversive properties of nicotine may be mediated through receptors containing this subunit, e.g. α4β2 nAChRs. Genetic studies in α5 and β2 nAChR-knockout mice also suggest that α5 and β2 containing nAChRs are involved in mediating the aversive properties of nicotine [33,34]. The specific nAChR that mediates the effect of large doses of nicotine on GAD67-GFP positive cells of SNCM is not known and whether receptors other than α4β2 nAChRs previously reported to be expressed by GABAergic cells, contribute to activation of these cells, remains unclear.

In the present study, GAD67-GFP positive cells were seen at several sites in the VTA including IP, SNR, RPC and SNCM and in close proximity to dopaminergic neurons. The exact contribution of SNCM GABAergic cells to the abuse-related aspect of nicotine is not known. However, GABAergic neurons are known to play an important role in regulating the activity of dopaminergic neurons of VTA and in modulating reinforcing effects of different drugs, including nicotine [8,35–37]. Dopaminergic neurons of VTA express both GABA_A_ and GABA_B_ receptors [38–40].

Optogenetic activation of VTA GABA neurons and local administration of GABA_A_ or GABA_B_ agonists to VTA decreases the burst firing of dopaminergic neurons and leads to decreased release of dopamine in Acb [17,35,41,42]. The GABAergic receptor agonist muscimol and the GABA_B_ receptor agonist baclofen both decrease nicotine self-administration [17,35,41] and baclofen attenuates nicotine-induced CPP [43]. GABA_B_ receptor agonists also reduce both the cue-induced reinstatement of nicotine-seeking [41] and the nicotine-induced reinstatement of extinguished nicotine-seeking [44].

The present neuroanatomical data demonstrating nicotine activation of GABAergic cells in VTA is in agreement with previous biochemical research reporting that acute single exposure to nicotine causes transient activation of GABAergic transmission in VTA. However, activation of these neurons by the high doses of nicotine which have, in other studies, shown to be aversive [21,22] implies that the GABA neurons in SNCM may be a component of a discrete circuitry that could mediate aversive responses to nicotine.

In summary, results of the present study identify a subpopulation of GABAergic cells in SNCM, in close apposition to dopaminergic cells of VTA, which are activated by an aversive dose of nicotine. It remains to be determined if the nicotine-activated GABAergic cells of SNCM contribute to the reward and/or the aversive abuse-related aspects of nicotine.
